# Understanding the Process of Acceptance Within the Nurse–Patient Therapeutic Relationship in Mental Health Care: A Grounded Theory

**DOI:** 10.3390/healthcare12222233

**Published:** 2024-11-08

**Authors:** Andrea Aznar-Huerta, Antonio R. Moreno-Poyato, Gemma Cardó-Vila, Teresa Vives-Abril, Juan M. Leyva-Moral

**Affiliations:** 1 Hermanas Hospitalarias, Hospital Mare de Deu de la Mercè, 08042 Barcelona, Spain; aaznarhuerta@gmail.com; 2 Department of Public Health, Mental Health and Maternal and Child Health Nursing, Faculty of Nursing, Universitat de Barcelona, 08036 Barcelona, Spain; 3 Grup de Recerca en Cures Infermeres de Salut Mental, Psicocials i de Complexitat, NURSEARCH—2021 SGR 1083, 08036 Barcelona, Spain; 4 Institut Neuropsiquiatría i Adiccions, Consorci Mar Parc de Salut, 08003 Barcelona, Spain; gcardo@psmar.cat; 5 Escola Superior Infermeria del Mar (ESIMar), Consorci Mar Parc de Salut, 08003 Barcelona, Spain; mtvivesa@gmail.com; 6 Department of Nursing, Faculty of Medicine, Universitat Autònoma de Barcelona, 08193 Barcelona, Spain; juanmanuel.leyva@uab.cat; 7 Grup de Recerca Infermera en Vulnerabilitat i Salut, GRIVIS—2021 SGR 120, 08036 Barcelona, Spain

**Keywords:** nurse–patient therapeutic relationship, mental health care, acceptance process, grounded theory, person-centered nursing

## Abstract

**Background/Objective:** Deepening knowledge about the meaning of acceptance in the context of the nurse–patient relationship will help us to understand the importance and value that people with mental health challenges attach to this attribute in the process of the therapeutic relationship. The objective of this study was to understand the process of acceptance as part of the therapeutic relationship between nurses and patients in the field of mental health. **Methods:** This study employed a qualitative research design using Strauss and Corbin’s grounded theory approach. Using theoretical sampling, data were collected through unstructured interviews with mental health nurses and with people with mental health challenges. **Results:** Acceptance is a complex and dynamic process that takes place when both patients and nurses feel and make the other feel like an individual human being. Acceptance is not an automatic process; rather, it requires awareness on behalf of both parties involved in the therapeutic relationship. The creation of a non-hierarchical caring environment is fundamental for fostering mutual acceptance and engagement in the therapeutic process. **Conclusions:** Nurses must develop self-awareness and reflect on their attitudes and biases to provide person-centered care. Nurse training and personal development are essential requirements for achieving an effective therapeutic relationship and empowering patients in their recovery process.

## 1. Introduction

Current international mental health policies focus on recovery-based approaches for people with mental health challenges [1,2]. The care provided by nurses should aim to treat the whole person, keeping them at the center of the care process [3]. The paradigm shift that has taken place in recent years has reduced the stigma towards the person with mental health challenges and their environment, as well as distancing us from the diagnostic label, which in turn allows for a patient empowerment effort that will result in a faster and more satisfactory recovery [4]. Therefore, it is clear that to provide person-centered, recovery-based care, it is necessary to have a greater knowledge and understanding of the care processes experienced by people with mental health challenges.

According to Peplau, nursing is defined as an interpersonal process, based on a human relationship where one of the parties is unwell and requires individualized care and where the other party, the nurse, must be able to recognize those altered needs and thus be able to provide an effective and efficient response [5]. Thus, the therapeutic relationship is a key tool in daily nursing practice, which includes attitudes and behaviors with a deontological implication, with special visibility in the field of mental health nursing [6]. It is an intervention in itself in which the nurse uses interpersonal skills as a process to assist the person in their empowerment with the goal of achieving wellness and recovery by collaborative working [7].

The establishment of the therapeutic relationship is therefore a dynamic and ever-changing process. Consequently, the goals and interests of both the nurse and the patient evolve as the process progresses [8]. Once the bond is created, the goals will become less divergent; at this point, the nurse begins to involve the person in their own health process and in their own care, thus carrying out an important empowerment task [9]. This relationship is essentially an expression of reciprocity between nurse and patient [10]. The suited establishment of a therapeutic relationship has been shown to not only improve health outcomes for the patient [11] but also reduce the levels of stress that the professional can endure [12]. In the context of mental health care, unlike other general care settings where the relationship may be more procedure-oriented, the nurse–patient relationship is particularly significant, as it requires specific communication skills and the ability to build a trustful bond [13]. Mutual respect, bidirectionality, positive reinforcement, acceptance, empathy, presence, connection, authenticity, trust, reciprocity, and individualization of care, among others, are key attributes identified in the literature for the development of an adequate therapeutic relationship [14]. Although these common factors in the therapeutic relationship go beyond the patient’s mental disorder [15], the therapeutic relationship can be affected depending on the mental health issue, as the patient’s needs may vary [16].

To create this therapeutic interpersonal relationship, the professional embarks on a process involving unconditional patient acceptance, putting aside their thoughts and ways of doing things [17]. From a theoretical perspective, the term unconditional acceptance stems from Rogers [18] who defines it based on psychological therapy, as the complete acceptance of the individual through their experiences and behavior, without any kind of critical evaluation or self-critical tendency. From the perspective of care, theories have also arisen about its construct; according to Watson in his theory of the intentionality of care, nurses must forget about prejudices and not impose their own standards, accepting the individual as the person they are and who they may come to be [19,20]. There is empirical evidence that confirms that the acceptance process is carried out without implementing any type of prejudice or personal idea regarding the patient’s situation, the decisions the person has made, or their behavior [21]. This requires an element of self-protection on behalf of the nurse with the objective of not suffering what is experienced during clinical practice beyond their professional life [22]. However, there is a lack of studies that present explicit results on the phenomenon of acceptance during the care process, and there are even less in the context of mental health nursing care.

Deepening knowledge about the meaning of acceptance in the context of the nurse–patient relationship will help us to understand the importance and value that people with mental health challenges attach to this attribute in the process of the therapeutic relationship. If we truly want to provide care based on agreement and supported decision-making, it is important to delve deeper into what this phenomenon means for all parties involved in the process, identifying both expectations and needs. This will help nurses to reformulate strategies and prioritize care. Consequently, this study set out to understand the process of acceptance in the context of the establishment of a therapeutic nurse–patient relationship.

## 2. Materials and Methods

### 2.1. Design

This study employed a qualitative research design using Strauss and Corbin’s grounded theory approach [23] to understand the meaning of acceptance within the therapeutic relationship process.

### 2.2. Study Setting and Recruitment

Mental health nurses living in Catalonia (Spain) were invited to participate using social media, institutional mailing lists, and personal contacts. The only inclusion criterion was to be working in mental health services at the time of the interview. Patients were invited to participate through information placed in mental health centers and by care teams. They were provided with basic information about the objectives and procedures, making it clear that participation was voluntary. Those who showed an interest were asked for permission to share their contact details with the principal investigator. A few days later, the principal investigator met individually with those who showed an interest, providing them with detailed written and oral information about this study and allowing them to clarify any possible doubts. The principal investigator contacted the nurses directly to provide them with comprehensive written and oral details of this study and to address any queries they may have had.

### 2.3. Inclusion and/or Exclusion Criteria

The inclusion criteria for patients were being hospitalized at the rehabilitation unit or day center for at least three months or being a user of outpatient adult mental health services for at least three months with regular nursing follow-up. This study excluded patients whose nurse in charge considered them to be psychopathologically unstable. Additionally, patients who were physically or medically restrained, at high risk of self-harm, under observation for a high risk of unexpected behavior, or those with language barriers were excluded. In line with grounded theory studies, the sample size was determined by theoretical data saturation, i.e., data were collected until the point that no new data arose from the interviews [24]. Nonetheless, the estimation was that 20–30 participants were to be interviewed.

### 2.4. Data Collection

Data were obtained by conducting unstructured interviews among a sample of mental health nurses and patients recruited using theoretical sampling [25]. This involved an iterative process of data collection and analysis where emerging concepts guided subsequent participant selection and data gathering, allowing us to refine and elaborate on developing theoretical categories until theoretical saturation was achieved [26].

Interviews lasted approximately 50 min and were held face-to-face in Spanish, in participants offices’ or using a video-conferencing platform (Microsoft Teams^®^ 2003). No substantial differences in data richness between face-to-face and online interviews were found. Recent research supports this observation, demonstrating that video-conferencing platforms can facilitate high-quality, in-depth qualitative interviews comparable to face-to-face interactions [27,28].

Data collection took place from January 2020 to April 2021 and was conducted by two nurses experienced in qualitative research. Before beginning the interview, participants provided informed consent through a detailed written form and a verbal explanation, and we ensured they fully understood this study by requiring that they explain it back in their own words. All possible questions were answered before signing the informed consent. Prior to conducting the interviews, the principal investigator created a brief inventory of open-ended questions, which were subsequently discussed with the research team. To ensure question clarity and relevance, these were piloted with a small group of eligible individuals who were not part of the final study sample. This process allowed for refinement of the questions and confirmed their appropriateness for the target population [29].

All interviews were digitally audio-recorded with participants’ permission and transcribed verbatim immediately after. Once the transcription was accurately verified, the audio files were deleted. The participants were invited to respond to broad, large, sweeping, and general questions, such as “What is acceptance for you?”, “What are the barriers and facilitators of acceptance?”, and “How does acceptance impact care and the patient-nurse relationship?” Other questions naturally emerged as interviews progressed, providing new insightful data. Field notes and memos were also collected to note potential areas for further study. The Consolidated Criteria for Reporting Qualitative Studies (COREQ) was used to guide the reporting of this process [30].

### 2.5. Data Analysis

To analyze the data, the constant comparative method was used with the support of NVIVO^®^ 12 software and was carried out simultaneously with data collection. After each interview, a self-debriefing exercise was carried out to summarize the discussions and identify possible areas of clarification, noting congruities and inconsistencies with other interviews, all of which are actions of great importance for the sampling process. The transcripts were independently reviewed several times by two researchers, who familiarized themselves with the content before starting open, axial, and selective coding [23]. Through a process of continuous critical reflection, 1561 initial codes were identified, which were organized into 34 subcategories and grouped into 3 categories with an interpretive meaning. No deviant cases were observed. Memo notes were of great importance in the process of capturing essential information for discussion, questions, and reflection. This was of particular significance in the generation of codes and categories, including properties and dimensions. Finally, a core category was identified and defined to understand the phenomenon under study.

Four factors helped to verify the appropriateness of core category: (a) Comprehensiveness: the core category integrated all categories and subcategories identified during analysis, providing a cohesive explanation of the studied process; (b) Theoretical sufficiency: the concept demonstrated depth and breadth in its properties and dimensions, indicating that theoretical saturation was achieved; (c) Explanatory power: the concept effectively clarified variations in the data and provided meaningful insights into the phenomenon under study; (d) Relevance: the concept was firmly grounded in participants’ experiences and addressed their main concerns [31].

### 2.6. Ethical Considerations

All participants were informed about the voluntary nature of their participation, their ability to withdraw from this study at any time without providing a reason, the confidentiality of their participation, and the deidentification of data for reporting. All recorded interviews were encrypted and stored on secure servers with restricted access. Personal identifiers were removed to ensure anonymity, and only authorized personnel could access the data. The Parc de Salut Mar Research Ethics Committee (No. 2019/8523/I) approved this study.

### 2.7. Rigor and Reflexivity

Data credibility and consistency were ensured by performing debriefing discussions at the end of each interview. Moreover, transcripts and the findings were also discussed with participants, wo affirmed the accuracy of the interpretation of their interview. Verbatim transcripts were translated into English and verified by two bilingual members of the research team, applying a previously informed process to ensure that the translations retained not only syntax but also theoretical meaning. The results were verified with participants and external consultation, with minor refinements for two category names. The core category was collectively noted to define the central meaning without further recommendations. To promote reflexivity throughout the process, the research team maintained an open dialogue with ample opportunities for self-reflection, open communication, and reflective practices, which resulted in the team gaining a deep understanding of the data.

## 3. Results

A total of 29 interviews were conducted, involving 15 patients (7 women) with a mean age of 45 years (range 25–54), and 14 nurses (10 women) with a mean age of 38.1 years (range 24–60). The main characteristics of the participants are summarized in Table 1.

**Figure 1:**
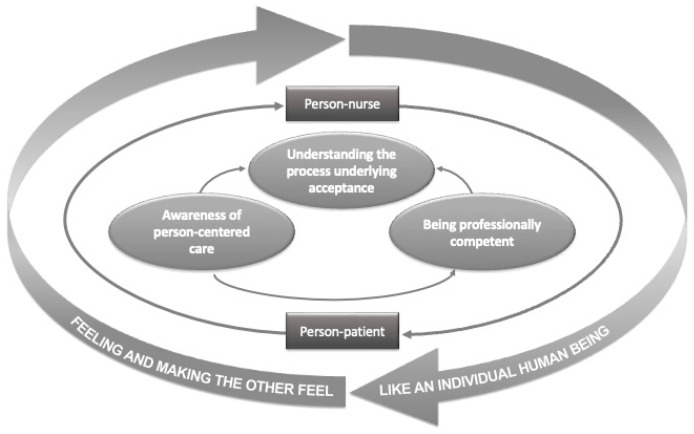
Diagram of the acceptance process.

A core category called “Feeling and making the other feel like an individual human being” was identified, which was informed by three categories: “Understanding the process underlying acceptance”, “Awareness of person-centered care”, and “Being professionally competent” (see Figure 1 and Appendix A).

### 3.1. Core Category: Feeling and Making the Other Feel Like an Individual Human Being

The data indicated that acceptance is a complex, living, and dynamic concept that is evidenced when both patients and professionals feel and make the other feel like an individual human being. For this purpose, it is necessary for both parties to realize that this is a process that does not evolve automaticall; rather, it requires the actors involved to perceive the need to put the person at the center of their attention and care. Awareness of the complexity of this process leads professionals and patients to realize that professional competence is an essential prerequisite.

### 3.2. Understanding the Process Underlying Acceptance

The data revealed that acceptance is not something that can be achieved spontaneously in the context of the nurse–patient relationship. Indeed, it is a unique process within each therapeutic relationship; i.e., each care encounter is unrepeatable as it arises in specific contexts where the personal and professional realities of the people involved in the therapeutic encounter are dynamic. Consequently, acceptance is explained as a living and changing process, characterized by its genuineness in the context of individualized care.

“…I think that you can achieve more when you manage to understand the person, right, when…It’s true that at the beginning it can be difficult because…you lack trust, you don’t know the person…but when…I think that, if you manage to understand this person well, to put yourself in the other person’s shoes…it can become easier.”

(Nurse 12)

“…(the exercise of accepting) I don’t think it’s uniform, I mean…for example, it depends on the situation that needs to be faced.”

(Patient 2)

Participants stated that acceptance is not a linear process that happens regularly and can be predicted; instead, that each therapeutic encounter is created and evolves in a unique way, which requires the authenticity of both actors and their commitment to the care process. Therefore, it is a complex process, which materializes in the mutual transcendental experience of feeling like a person and making the other feel like a person. It should be noted that the data showed that this complexity is bidirectional, i.e., what happens to patients affects professionals and vice versa. Finally, the data indicated that acceptance is a process influenced by the context and its characteristics, which can range from local structural aspects to organizational, political, or economic aspects.

“Of course, the context issue is what I was talking about before, whether you want it or not, its limiting, these are things that don’t let you…”

(Nurse 4)

“…so to speak, in this relationship there is a part that depends on the person and another that depends on the nurse.”

(Patient 13)

### 3.3. Awareness of Person-Centered Care

This category refers to the fact that to speak of acceptance is to regard patients and professionals as an indivisible dyad, the essence of which lies in the experience of feeling like an individual human being and making the other party feel so too. The participants stated that the evolution of mental health in recent decades, both at the health and social levels, has led to a paradigm shift that places the person at the center of care, which demands an increase in their participation in the therapeutic process itself.

“I think it has changed a lot, now we can see the sick people, before we didn’t weren’t visible…Before you were like a—like a number, you know? You felt like a number. Now you don’t feel like a number, nor a label, you feel like a person.”

(Patient 7)

“Yes, I didn’t feel like a number or patient x, I felt like myself.”

(Patient 11)

The data showed that to implement this new paradigm, it is necessary to establish an egalitarian, one-to-one relationship, free of preconceived ideas, attitudes, and/or stigmatizing behaviors. A lack of judgment, regardless of the emotional or cognitive moment in which the person is, is key to establishing a bond of trust that enables the design of an individualized and consensual therapeutic plan between both parties.

“I think that making decisions is also important, but I think that we must make them together…yes, between both of us. For example, I can give my opinion, she can give hers, and we can be like…well, of course, you are the professionals, you are the ones who know, I can give my opinion, but I can be wrong…”

(Patient 14)

“…I have also perceived that, for example, I could tell you…they have treated me as…as an equal, haven’t they? And in a way this is part of what I was saying about complicity, isn’t it?”

(Patient 2)

Regarding the nurse–patient bond, the participants mentioned that this is established between both parties through a rapprochement, an approach, and a deep knowledge of those involved through therapeutic encounters that take place over time. From the patients’ perspective, not feeling judged favors feeling like a person and, therefore, respected and recognized with one’s own idiosyncrasies; this allows nurses and patients to become actively involved in the relationship established with the professional and in the care process. Therefore, placing the person at the center of care is achieved when both parties are willing to commit to it.

“It felt good to me to be given time, she [the nurse] was the one who gave me the space. Weeks went by, a week and a half or two and she came around to me.”

(Patient 4)

“…, and if not, then tomorrow, and if not…because he comes next week and asks me, but I leave him his space.”

(Nurse 3)

“For me it’s very important that he accepts me as a person, that he respects me, that he values me, that he considers me, that he listens to me, yes.”

(Patient 14)

### 3.4. Being Professionally Competent

According to the participants, professional competence is essential for establishing person-centered care and requires knowledge, skills, attitudes, experiences, and techniques that are acquired, worked on, and continuously improved. In their opinion, the competent mental health nurse is one who, in a friendly manner, is able to place the individual at the center of the care process by getting to know the person, accepting them, without judgment, and displaying a receptive attitude with patience and humility, as well as being able to listen without judging—in other words, making them feel that they are an individual human being.

“The way she treats you and all; always trying to help. Her kindness, her patience…I’m very comfortable with her. Her professionalism, she cares a lot.”

(Patient 9)

“Yes, exactly, she has been very consistent, very repetitive with the same things, and it has made me realize this. The truth is that her attitude, apart from being good, is very kind, which is important.”

(Patient 11)

“…we need compassionate, empathetic, active-listening and expert nurses.”

(Nurse 4)

The participants also stated that it is necessary for nurses to show initiative, proactivity, and the ability to adapt care to each individual, to the moment they are in, and to the context in which it is taking place. It is essential to listen, know, and understand the other through communication, active listening, understanding, empathy, and compassion. One of the characteristics of competent mental health nurses is their willingness, accessibility, and availability to patients at all times.

“For me, it’s important that you feel listened to, because based on what you tell them, they will act accordingly.”

(Patient 3)

“Yes, it’s the intention to give you something, for you to receive something in some way.”

(Patient 10)

“In the sense of awareness of what I am going to do, I think that yes, obviously, there is a basis, there has to be a personal basis of personality, that you may be one type of person or another, but I think there must be a basis of awareness of what you are going to do, which is complicated, right?”

(Nurse 4)

“…you have these kinds of things, life is like that, you have to do these kinds of things to feel better, we give you tools, we give you support and at any time you can write to us when you are no longer in the day hospital.”

(Patient 11)

Finally, the participants affirmed that nursing competence requires self-knowledge, introspection, and self-protection. This is necessary in order to provide individualized care that enables limitations to be assessed and the care plan to be redirected when necessary for the patient’s well-being. Ultimately, this acceptance makes the nurse aware of the person and their needs.

“To address what you’re asking for…your needs.”

(Patient 5)

“…that my needs are met in the sense that I am here for a number of reasons.”

(Patient 1)

“You don’t connect with all experiences in the same way, although you try, don’t you, because with empathy you always try to put yourself in the other person’s shoes, but it’s true that there are certain experiences that you connect with more than others. And that makes you gain some things or others.”

(Nurse 5)

## 4. Discussion

This study indicates that unconditional acceptance is a complex, living, and dynamic process that is evidenced when both patients and professionals feel and make the other feel like an individual human being. This conceptualization is fully in line with the “person-centered care” approach since it places the person at the center of the therapeutic process with a holistic approach based on an egalitarian, one-to-one relationship, without hierarchy and respecting human rights [32].

The results indicate that, for both patients and nurses, acceptance emerges as part of a single process between two people as an indivisible dyad. Consequently, it is essential for the professional to create a caring and relational environment that enables the creation of a therapeutic relationship making the other person feel like an active participant in their own treatment [20]. Therefore, generating spaces of care without therapeutic hierarchy is fundamental to ensuring that the individual collaborates in the process, which enables personal empowerment by designing an individualized therapeutic plan agreed upon by both parties [33]. As a process that is part of the nurse–patient relationship, it is dynamic and bidirectional; therefore, it is conditioned by the context of care [6] and, obviously, by the situation of the other person [14,34].

Another aspect highlighted in the results was the importance of commitment, both on behalf of nurses and patients, as a necessary element in the acceptance process. This finding is in line with other studies that explored therapeutic engagement in mental health nursing care, both in a community setting [35] as well as in involuntary hospitalization settings, where perspectives held by patients and nurses are often more distant [36,37,38].

While the results confirm the bidirectionality of the acceptance process, both patients and nurses emphasized the importance of the nurse’s professional competence for an effective acceptance process [39]. Professional competence is essential to establish a quality therapeutic relationship. Both patients and nurses emphasized that nurses must have skills and techniques that are acquired, worked on, and continuously improved in order to relate with and accept others. One of the characteristics of competent mental health nurses is their willingness, accessibility, and availability to patients at all times [40]. Likewise, the ability to accept the patient also requires self-knowledge, introspection, and self-protection [9,41,42]. Acceptance of the other allows nurses and patients to feel and connect with themselves, facilitating patients in their recovery process and protecting nurses from burnout [43].

### 4.1. Strengths and Limitations

This study has several strengths and limitations that should be kept in mind when interpreting the results. A strength of this study is that it contributes to the limited knowledge base on the process of acceptance in the nurse–patient relationship. Furthermore, the involvement of participants in the verification of the findings aligned this study with the lived experience and leadership movements. Although not strictly a limitation, in this study, 14 nurses and 15 patients participated from the mental health care setting of Catalonia. The method used sought to understand their experiences and cannot be assumed to have produced findings that explain the experiences of the entire population linked to mental health care services. However, this study captures the perspectives of both nurses and patients from different care settings. Moreover, given the method used, the results may lead to a deeper and more nuanced understanding of the phenomenon studied. Nevertheless, more research is needed in this area.

### 4.2. Implications for Policy and Practice

Mutual acceptance between nurses and patients must be fostered through a conscious, person-centered process. Therefore, nurses must recognize the relevance of establishing a close, egalitarian, and nonjudgmental relationship with patients. This may improve the quality of care and increase therapeutic engagement. Consequently, it is critical for nurses to acquire and maintain a high level of competence in caring for patients with mental health problems. Training should include both technical skills and a focus on self-awareness and personal development, which will allow for a deeper understanding of the patient’s needs. In this regard, nurses should be encouraged to reflect on their own attitudes and biases, as well as their interactions with patients. This will help to improve the quality of care and the therapeutic nurse–patient relationship. Along these lines, a sense of self-responsibility and reflective practice should be promoted among the general population regarding their role in the processes of care and the relationship with health professionals. Finally, it is necessary to involve organizations, managers, and health care decision-makers to promote strategies, measures, and resources to generate contexts that facilitate a quality nurse–patient relationship.

## 5. Conclusions

This study contributes to our understanding of the acceptance process within nurse–patient relationships in mental health settings. The findings suggest that acceptance emerges as a reciprocal phenomenon, characterized by the mutual recognition and validation of each other’s individuality and humanity. This study has shown that the process of mutual acceptance between nurses and patients with mental health challenges is complex and requires commitment from both parties. It is important that an egalitarian and non-judgmental relationship is established, considering the context and individual characteristics of each person. In addition, the professional competence of nurses is necessary to ensure a successful process, and this competence requires self-knowledge and continuous improvement.

## Figures and Tables

**Table 1:** Participant characteristics.

Patients (n = 15)	
Gender	
Male	8
Female	7
Mean age	45
Mean years living with mental disorder	17.67
Mental health service (user)	
Mental Health Hospital	7
Community Mental Health Services	8
**Nurses (n = 14)**	
Gender	
Male	4
Female	10
Mean age	38.1
Area of expertise	
Mental Health Hospital	8
Community Mental Health Services	6
Mental health specialization	
Yes	7
No	7

## Data Availability

The raw data supporting the conclusions of this article will be made available by the authors on request.

## Appendix A

**Table A1:** Categories of the acceptance process.

Core Category	Category	Sub-Category
Feeling and making the other feel like an individual human being	Understanding the process underlying acceptance	Unique, two-way process
		Characterized by genuineness
		Unique, changeable
		Includes individualization
		Singularization
		Mediated/conditioned by context
		Binding (mutual involvement)
		Slow
		Progressive
		Fluctuating
	Awareness of person-centered care	Both professionally and socially
		Making the other person feel like a person
		Being part of the process
		Pathology
		State of health
		Emotional state
		Cognitive state
		Protection/security/reassurance
		Context
	Being professionally competent	Professional competence
		Non-judgmental/labeling/judging
		Preconceived ideas/stigma/neutral
		Professionalism
		Listening
		Accessibility
		Introspection
		Knowledge
		Understanding
		Knowledge of the other/understanding
		Compassion/empathy
		Self-knowledge/self-protection
		Boundary setting/managing/redirection
		Proactivity
